# An HDAC in the Cytoplasm, not the Nucleus, Plays a Pathogenic Role in Huntington's Disease

**DOI:** 10.1371/journal.pbio.1001718

**Published:** 2013-11-26

**Authors:** Richard Robinson

**Affiliations:** Freelance Science Writer, Sherborn, Massachusetts, United States of America

[Fig pbio-1001718-g001]The gene for Huntington's disease (HD) was discovered in 1993 and ever since has been puzzling researchers intent on understanding its effects. The mutation, an expanded CAG repeat, is translated into an extended polyglutamine tract in the huntingtin protein (HTT), which leads to protein misfolding, accumulation of sticky protein aggregates in both cytoplasm and nucleus, and degeneration of neurons, first in the brain's striatum and later in the cortex and elsewhere. There are no disease-altering treatments and no single hypothesis of disease pathogenesis. In a new study, Michal Mielcarek, Gillian Bates, and colleagues elucidate a key role for the transcription regulator histone deactylase 4 (HDAC4)—not within the nucleus as you might expect, but instead in the cytoplasm.

**Figure pbio-1001718-g001:**
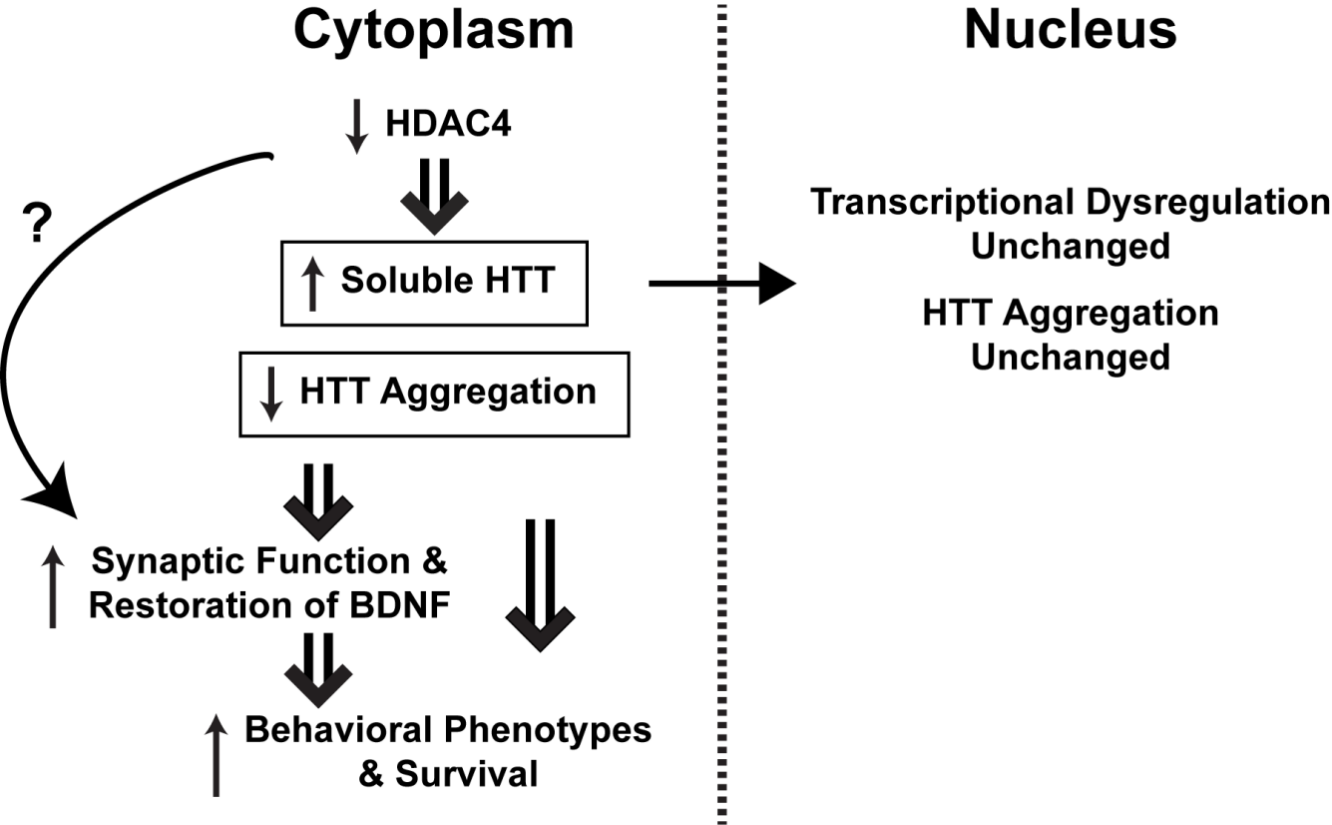
HDAC4 acts in the cytoplasm of brain cells to exacerbate the Huntington's disease (HD) pathogenic process. Reduction of HDAC4 levels ameliorates cytoplasmic-related HD phenotypes and improves survival, but does not change gene transcription.

One prominent feature of HD molecular pathology is a global transcriptional dysregulation, likely driven in part by reduced histone acetylation. To counterbalance that effect, researchers have investigated whether inhibiting HDACs might be therapeutic, and one HDAC inhibitor has produced promising results in preclinical trials in models of HD.

There are 11 different mammalian HDACs, and in order to better characterize their individual contributions to HD, the authors examined the effects of partially or completely knocking out each one. They found that reducing HDAC4 by 50% in a mouse model of HD reduced neuronal dysfunction of striatal neurons and delayed loss of motor function of the mice, allowing them to perform a balancing task at 12 weeks of age as well as untreated mice who were a month younger. Treatment also extended lifespan by about 20%, a significant improvement in this aggressive disease model of HD.

Antibodies against HDAC4 and either mutant or wild-type HTT indicated that HDAC4 bound to mutant HTT in either soluble or aggregated form, but not to normal protein. Like HTT, HDAC4 includes a polyglutamine region, and it is likely that its interaction with mutant HTT is through the reciprocal attractions between these regions. Reducing HDAC4 reduced the total burden of HTT aggregates throughout the brain while increasing the amount of soluble HTT, indicating that treatment delayed the aggregation process. The work thereby finds a novel route to modulating the toxicity of HTT.

HDAC4 remains sequestered in the cytoplasm until it is called upon to shuttle into the nucleus to take part in regulating transcription. Co-labeling HDAC4 and mutant HTT indicated that they localized together in the cytoplasm, but not the nucleus, and that reducing HDAC4 reduced aggregation in the cytoplasm while leaving the number of nuclear aggregates unchanged. This ability of the work to separate cytoplasmic pathologies from nuclear ones is an important advance in this field.

Surprisingly, reducing HDAC4 had no effect on the widespread transcriptional dysregulation that led HD researchers to consider HDAC inhibitors in the first place. It did, however, have one potentially important effect on gene expression: it largely restored the levels of brain-derived neurotrophic factor, a growth factor that neurons need for survival and that is known to be lost in HD. This effect is likely mediated through a cytoplasmic, not nuclear, mechanism, based on previous work on this pathway in HD; further work will be needed to understand it in detail.

These results have several implications for understanding and treating HD. There is no shortage of hypotheses of pathogenic mechanisms in HD, and this new mechanism is unlikely to be the sole cause of neuronal damage. But the discovery of a strictly cytoplasmic pathogenic effect of mutant HTT is new and will likely bring more attention to the cytoplasm as a site for further research. The demonstration that shifting the balance of mutant protein from aggregated to soluble forms has a beneficial effect further informs a long-standing debate in disorders of protein misfolding, about whether aggregates are toxic or protective, and will likely accelerate exploration of therapies to promote disaggregation. As mentioned, there are currently no disease-modifying therapeutics available for HD. The discovery that reducing HDAC4 has therapeutic effects in the HD model tested here—which happens to be a gold standard model in which most therapeutics fail when tested—will spur efforts to mimic this effect with small molecules to obstruct the interaction of HDAC4 and mutant HTT, or antisense therapies designed to prevent production of HDAC4 protein.

Mielcarek M, Landles C, Weiss A, Bradaia A, Seredenina T, et al (2013). HDAC4 Reduction: A Novel Therapeutic Strategy to Target Cytoplasmic Huntingtin and Ameliorate Neurodegeneration. doi:10.1371/journal.pbio.1001717

